# Multi-Stage Fitness Test Performance, $\dot{\text{V}}$O_2_ Peak and Adiposity: Effect on Risk Factors for Cardio-Metabolic Disease in Adolescents

**DOI:** 10.3389/fphys.2019.00629

**Published:** 2019-05-31

**Authors:** Karah J. Dring, Simon B. Cooper, John G. Morris, Caroline Sunderland, Gemma A. Foulds, Alan Graham Pockley, Mary E. Nevill

**Affiliations:** ^1^Department of Sport Science, Sport, Health and Performance Enhancement Research Centre, School of Science and Technology, Nottingham Trent University, Nottingham, United Kingdom; ^2^John van Geest Cancer Research Centre, School of Science and Technology, Nottingham Trent University, Nottingham, United Kingdom

**Keywords:** low-grade chronic inflammation, insulin resistance, cardio-metabolic disease, multi-stage fitness test, VO_2_ peak, adiposity

## Abstract

The role of physical activity in determining the metabolic health of adolescents is poorly understood, particularly concerning the effect on low-grade chronic inflammation (chronic elevation of pro-inflammatory cytokines IL-1β, IL-6, TNF-α and acute phase protein CRP, which is implicated in the etiology of atherosclerosis) and anti-inflammatory mediators such as IL-10. Furthermore, there is limited information on the mediating effects of performance on the multi-stage fitness test (MSFT), $\dot{\text{V}}$O_2_ peak and adiposity on risk factors for cardio-metabolic disease in adolescents.

**Purpose:** To examine the effect of performance on the MSFT, $\dot{\text{V}}$O_2_ peak and adiposity on risk factors for cardio-metabolic diseases in adolescents.

**Methods:** Following ethical approval, 121 adolescents (11.3 ± 0.8 year) completed the study. Risk factors for cardio-metabolic disease (circulating inflammatory cytokines, blood glucose and plasma insulin concentrations) was assessed using a fasted capillary blood sample. Participants were separated into quartiles based upon distance ran during the MSFT, the blood lactate response to submaximal exercise, $\dot{\text{V}}$O_2_ peak (determined during an uphill graded treadmill test), and adiposity (determined as the sum of four skinfolds). The blood lactate response to submaximal exercise and V0_2_ peak were measured in a sub-group of participants. Data were analyzed using two-way between-subjects ANCOVA and multiple linear regression.

**Results:** Participants with the lowest performance on the MSFT had higher blood concentrations of IL-6 (3.25 ± 0.25 pg mL^-1^) and IL-1β (4.78 ± 0.54 pg mL^-1^) and lower concentrations of IL-10 (1.80 ± 0.27 pg mL^-1^) when compared with all other quartiles (all *p* < 0.05). Yet, when categorized into $\dot{\text{V}}$O_2_ peak quartiles, no differences existed in any of the inflammatory mediators (all *p* > 0.05). Performance on the MSFT was the only predictor of IL-6 (β = -0.291, *p* = 0.031), IL-1β (β = -0.405, *p* = 0.005), IL-10 (β = 0.325, *p* = 0.021) and fasted blood glucose (β = -0.545, *p* < 0.001) concentrations. Adiposity was the only predictor of plasma insulin concentration (β = 0.515, *p* < 0.001) and blood pressure (diastolic: β = 0.259, *p* = 0.042; mean arterial pressure: β = 0.322, *p* = 0.011).

**Conclusion:** Enhanced performance on the MSFT, but not $\dot{\text{V}}$O_2_ peak, was associated with a favorable inflammatory profile in adolescents; whilst adiposity adversely affected plasma insulin, diastolic and mean arterial blood pressure. These findings demonstrate that enhancing performance on the MSFT and maintaining a healthy body composition are a potential therapeutic intervention for the attenuation of risk factors for cardio-metabolic diseases in adolescents.

## Introduction

Low-grade chronic inflammation is a key risk factor in the pathogenesis of cardio-metabolic diseases (including hypertension, hyperglycemia and early insulin resistance) and atherosclerotic plaques (Balagopal et al., 2011). The presence of low-grade chronic inflammation is currently the strongest predictor of cardiovascular events in adults, bettering traditional markers of dyslipidemia and hypertension (Petersen and Pedersen, 2005). Although cardiovascular disease typically presents during adulthood, the prevalence of low-grade chronic inflammation in adolescents (Balagopal et al., 2011) is of concern, as early and continued exposure increases the risk of early onset cardiovascular disease and type 2 diabetes (Gleeson et al., 2011).

Low-grade chronic inflammation is a chronic, 2- to 3- fold elevation in the concentrations of inflammatory mediators, including interleukin-1β (IL-1β), interleukin-6 (IL-6), interleukin-1 receptor antagonist (IL-1ra), tumor necrosis factor-α (TNF-α) and the acute phase protein C-reactive protein (CRP) (Petersen and Pedersen, 2005). Acute bouts of physical activity are implicated in the prevention of low-grade chronic inflammation through the anti-inflammatory response that occurs post-exercise (Gleeson et al., 2011). Recently, it has been shown that acute bouts of games-based activity transiently increased concentrations of anti-inflammatory mediators IL-10 and IL-1rα in healthy young people (Dring et al., 2019) and middle-aged men (Mendham et al., 2015). Increased concentrations of IL-10 and IL-1ra are reported to inhibit the synthesis of pro-inflammatory cytokines (IL-1β and TNF-α) and improve insulin sensitivity when assessed *in vitro* (Gleeson et al., 2011). Furthermore, regular participation in physical activity prevents excessive adiposity (Van der Heijden et al., 2012) and reduces adiposity in overweight adolescents (Rey et al., 2017) and adults (Alrushud et al., 2017). Although such findings support regular moderate intensity physical activity as a potential therapeutic intervention that protects against the development of risk factors for cardio-metabolic disease, the chronic effects of regular training resulting in enhanced physical fitness on low-grade chronic inflammation in adolescents are relatively unknown.

When assessing the effect of physical fitness on low-grade chronic inflammation a comprehensive range of inflammatory mediators (IL-1β, IL-6, TNF-α, and CRP) should be measured (Petersen and Pedersen, 2005). Yet, in adolescents and adults, research has focused on the relationship between physical fitness and a limited number of pro-inflammatory mediators (IL-6, TNF-α, and CRP) (Platat et al., 2006; Ischander et al., 2007; Bugge et al., 2012; Buchan et al., 2015). In adolescents, the findings of previous studies assessing the relationship between physical fitness and inflammatory mediators IL-6, TNF-α and CRP are inconclusive with no apparent relationship (Platat et al., 2006; Steene-Johannessen et al., 2013), or inverse associations observed (Bugge et al., 2012; Silva et al., 2014; Buchan et al., 2015). Furthermore, the relationship between physical fitness and concentrations of anti-inflammatory mediator IL-10 is unknown despite the potential of IL-10 to reduce low-grade chronic inflammation and improve insulin sensitivity (Petersen and Pedersen, 2005). Increasing adiposity reduces the expression of IL-10 in normal weight and overweight individuals (Esposito and Giugliano, 2004; Utsal et al., 2013), whereas the effect of physical fitness on IL-10 concentration has only been studied once, in healthy, normal weight adults (Jürimäe et al., 2017a) and once in pubertal girls (Jürimäe et al., 2017b). Jürimäe et al. (2017a) reported no relationship between maximal oxygen uptake and IL-10 concentration in well-trained adult rowers. These null findings might relate to the well-trained study population, in that the variability of fitness among the participants was not diverse enough for a relationship to be established. However, when comparing well-trained, female adolescent rhythmic gymnasts against untrained counterparts, there was still no difference across 12 markers of inflammation, which included anti-inflammatory mediator IL-10 (Jürimäe et al., 2017b). Whilst the inflammatory profiles of the trained gymnasts and the untrained controls were similar, there was no measurement of physical fitness or body composition in the pubertal girls; therefore, the relationship between physical fitness, inflammatory markers and IL-10 concentration remains unknown, particularly in young people.

In previous studies in adolescents and adults, the effect of long-term training on risk factors for cardio-metabolic disease has been determined by peak oxygen consumption when using graded treadmill tests (Ischander et al., 2007; Bugge et al., 2012; Silva et al., 2014) and graded cycle ergometer tests (Steene-Johannessen et al., 2013). The discrepant findings of previous research could relate to the limitations of $\dot{\text{V}}$O_2_ peak as a measure of physical fitness (Coyle et al., 1983), as $\dot{\text{V}}$O_2_ peak is considered to be relatively insensitive to changes in training status, with up to 50% of an individual’s $\dot{\text{V}}$O_2_ peak being determined by genetics (Bouchard, 2012). Regular participation in moderate-to-vigorous activity moderates an individual’s exercise capacity and is the mechanism that stimulates the transient inflammatory response that prevents low-grade chronic inflammation (Dring et al., 2019). When focusing on the relationship between physical fitness and risk factors for cardio-metabolic disease the measurement of fitness should therefore be sensitive to changes in an individual’s ability to perform prolonged exercise (Strasser and Burtscher, 2018). The blood lactate response to submaximal exercise is more sensitive to changes in training status than maximal oxygen uptake in both adults (Edwards et al., 2003) and young people (Grant, 2001). Furthermore, the submaximal nature of the test allows the assessment of a heterogeneous population and therefore allows the comparison of individuals from sedentary, recreationally active and well-trained backgrounds. Performance on the MSFT is also a commonly used, reliable and easy to administer, field measure of physical fitness in young people (Ortega et al., 2008) and is sensitive to changes in training status (Aziz et al., 2005). Therefore, the blood lactate response to submaximal exercise and the MSFT are potentially better suited for examining the relationship between physical fitness (physical capacity to perform prolonged exercise) and risk factors for cardio-metabolic disease.

As excessive adiposity mediates an increase in low-grade chronic inflammation, several studies have assessed the relationship between different measures of body composition and levels of the pro-inflammatory mediators IL-6 and TNF-α (Galcheva et al., 2011; Bugge et al., 2012; Utsal et al., 2013; Lopez-Alcaraz et al., 2014). Findings are inconclusive in that adiposity has been reported to have no effect on the pro-inflammatory mediators in several studies (Steene-Johannessen et al., 2013; Lopez-Alcaraz et al., 2014). However, increased adiposity has been associated with higher IL-6 and TNF-α concentration in adolescents in other studies (Galcheva et al., 2011; Bugge et al., 2012; Utsal et al., 2013). Of the studies that have examined adiposity, only one has considered the potential mediating effects of physical fitness (Silva et al., 2014). In the study of Silva et al. (2014) maximal oxygen uptake test was the best predictor of metabolic risk (calculated from traditional risk factors including blood pressure and dyslipidemia). Although these findings suggest that physical fitness is important for the prevention of traditional cardio-metabolic risk factors, it remains unknown whether physical fitness or adiposity best predicts, or whether these variables additively predict, risk factors for cardio-metabolic disease in adolescents.

Therefore, the aim of the present study was to determine the effect of MSFT performance, the blood lactate response to submaximal exercise, $\dot{\text{V}}$O_2_ peak and adiposity on a comprehensive panel of pro- and anti-inflammatory cytokines in conjunction with traditional cardio-metabolic risk factors in adolescents. A secondary aim of the study was to determine whether peak oxygen uptake (also influenced by genetics), MSFT performance or blood lactate concentration during sub-maximal exercise (better markers of the capacity to perform prolonged exercise) or adiposity better predict risk factors for cardio-metabolic disease in adolescents.

## Materials and Methods

### Participant Characteristics

A cross-sectional sample of 140 adolescents aged 10–12 years were recruited to participate in the present study. Given that 19 participants withdrew from the study (*n* = 10 due to illness, *n* = 5 due to injury and *n* = 4 due to reluctance to provide a capillary blood sample), 121 young people (61 male, 60 female, age 11.3 ± 0.8 year) participated. All participants underwent anthropometric measures of body mass, height and sitting stature to predict age at peak height velocity (APHV, calculated using the method described in Moore et al., 2015), as the preferred measure of maturation. Body mass was measured using a Seca 770 digital scale which is accurate to 0.1 kg (Seca, Hamburg, Germany), and height was measured using a Leicester Height Measure which is accurate 0.1 cm (Seca, Hamburg, Germany), to allow the determination of body mass index BMI, [calculated as body mass (kg)/stature (m)^2^]. Participant characteristics were; height 151.9 ± 7.2 cm, body mass 43.1 ± 9.5 kg, BMI Percentile 52.3 ± 29.3; years from peak height velocity 1.9 ± 0.7 year (males -2.0 ± 0.7 year; females -1.9 ± 0.8 year).

### Study Design

Ethical approval was received from the Nottingham Trent University’s Ethical Advisory Committee (SPOR-400). Participants were recruited from local secondary schools and sports clubs in the East Midlands, United Kingdom. Written parental consent and verbal child assent was obtained during recruitment. Health screen questionnaires were completed by the participants’ parent/guardian and checked by a lead investigator to ensure there were no medical conditions that might affect participation in the study.

All trials were separated by a minimum of 7 days (further details of which are provided below). The field measurements (completed during the first trial) consisted of anthropometric measures (body mass, stature and sitting stature), skinfolds and the MSFT, in that order. The health measurements (completed during the second trial, which commenced at ∼8.30 am) consisted of resting blood pressure followed by a resting capillary blood sample (fasted from 9 pm the previous evening). Finally, a sub-sample of participants (68 participants, 30 male, age: 11.6 ± 0.6; APHV: -1.9 ± 0.7 year) completed exercise laboratory tests including a submaximal treadmill test and a $\dot{\text{V}}$O_2_ peak test, which were separated by 20 min passive recovery. Only a sub-sample of participants from the study population volunteered to complete the final part of the study. Those that removed themselves from the exercise laboratory tests did so as they were not willing to take an additional day off school. Prior to all measurements, participants were asked to refrain from moderate-to-vigorous physical activity for 24 h. A telephone call was made to parents/guardians the evening prior to the testing sessions to ensure compliance with the study requirements.

### Field Measures

#### Body Composition

Skinfold thickness was measured using a Harpenden Caliper (Baty International, Burgess, Hill, United Kingdom) at four sites (tricep, subscapular, supraspinale, front thigh). All measurements were taken twice in rotation and on the right-hand side of the body. An average of the two measurements was taken unless the difference between the two measurements was >5%. In this circumstance, a third measurement was taken and the median value used as the criterion measure. All skinfold measures were completed by trained kinanthropometrists using methods described in The International Society for the Advancement of Kinanthropometry (2001). The use of skinfolds in assessing body composition in young people is reported as an effective, valid and reliable method in young people (Yeung and Hui, 2010; Bugge et al., 2012). Specifically, the sum of the four skinfold thickness scores was the preferred assessment of body composition in the present study, as estimating body fat percentage from skinfold thickness has been associated with large random error and significant systematic error (Reilly et al., 1995).

#### Multi-Stage Fitness Test (MSFT)

During the MSFT, participants completed progressive 20-m shuttle runs until the point of volitional exhaustion (Ramsbottom et al., 1988). The MSFT started at a speed of 8.5 km h^-1^ and increased by 0.5 km h^-1^ for each 1-min stage completed. Participants were fitted with a heart rate monitor (First Beat Technologies Ltd., Finland) prior to the start of the test and heart rate was monitored in real-time throughout its duration. Verbal encouragement was provided throughout to ensure participants worked to the point of volitional exhaustion. The distance ran during the MSFT was used as the criterion measure.

### Health Measures

#### Blood Pressure

On arrival at the exercise laboratory following an overnight fast, participants were seated quietly for 5 min prior to the measurement of blood pressure. Two blood pressure measurements were taken from each participant’s left arm, which was rested at chest height, using an HBP-1300-United Kingdom sphygmomanometer (Omron, Milton Keynes, United Kingdom). The average of the two blood pressure measures was used as the criterion measure. If systolic blood pressure differed by >5 mmHg, then a third blood pressure measurement was taken and the median value used as the criterion measure. Mean arterial blood pressure was determined using the following calculation (Smeltzer et al., 2010): *diastolic blood pressure + {[0.33*^∗^ *(systolic blood pressure – diastolic blood pressure)]}*.

#### Capillary Blood Samples

Capillary blood samples were obtained early in the morning following an overnight fast and during the speed lactate treadmill test (baseline and following each progressive stage). Prior to the fasted capillary blood sample, participants’ hands were warmed by submersion in warm water with clothing placed over their chosen arm to increase capillary blood flow. A Unistik single-use lancet (Unistik Extra, 21G gauge, 2.0 mm depth, Owen Mumford Ltd., United Kingdom) was used and blood was collected into three 300 μl EDTA microvettes (Sarstedt Ltd., United Kingdom). A 25 μl whole blood sample was collected using a plain pre-calibrated glass pipette (Hawksley Ltd., United Kingdom) and immediately dispensed into 250 μl of ice-cooled 2.5% v/v perchloric acid to be deproteinised. The whole blood samples and the diluted perchloric acid samples were centrifuged at 1500 × *g* for 5 min (Eppendorf 5415C, Hamburg, Germany). Plasma was pipetted from the original whole blood samples and placed into one of three 500 μl plastic vials for subsequent analysis. All samples were immediately frozen at -20°C and transferred to a -80°C freezer at the earliest opportunity.

Blood glucose concentrations were determined in duplicate using a commercially available assay (GOD/PAP method, GL364, Randox, Ireland) and were read spectrophotometrically (intra-assay coefficient of variation (CV) = 2.3%). Plasma insulin concentrations were determined using a commercially available ELISA (Mercodia Ltd., Sweden; CV = 3.2%). Fasted blood glucose and plasma insulin concentration were used to calculate the HOMA index (fasting plasma insulin (μU mL^-1^) x fasting blood glucose (mmol L^-1^)/22.5), as a measure of insulin resistance in adolescents (Keskin et al., 2005). Pro-inflammatory (IL-1β, IL-6, TNF-α) and anti-inflammatory (IL-10) cytokine concentrations were determined using an AimPlex, flow cytometry-based multiplex immunoassay (YSL Bioprocess Development Company, Pomona, United States) and a Beckman Coulter Gallios^TM^ flow cytometer and Kaluza^TM^ acquisition and analysis software (Beckman Coulter, London, United Kingdom). CRP concentrations were determined using the same approach, but on a separate plate. The intra-assay CV based on eight repeat measurements for inflammatory cytokines were as follows: IL-6: 15.9%, IL-1β: 17.4%, TNF-a: 14.7%, IL-10: 13.2% and CRP: 10.4%. Blood lactate concentrations were determined in duplicate using a commercially available assay (PAP method, LC2389, Randox, Ireland) and were read spectrophotometrically (CV = 6.7%).

### Exercise Laboratory Measures

#### Blood Lactate During Sub-Maximal Exercise

A sub-sample of participants completed a submaximal test on a calibrated treadmill (Technogym, Italy). Prior to participation, participants were fitted with a heart rate monitor (First Beat Technologies Ltd., Finland) and maximum heart rate during the final minute of each stage was recorded. Participants completed three to six, 4-min runs, interspersed with 1-min rest whilst a capillary blood sample was taken. The first stage of the test was completed at an individualized speed that was comfortable for the participant (between 6 and 8 km^-1^ h^-1^), which increased by 1 km^-1^ h^-1^ for each stage completed thereafter. The blood lactate concentration at 8.5 km h^-1^ was used as the criterion measure and was calculated by mathematically fitting a curve to the blood lactate-running speed relationship.

#### $\dot{\text{V}}$O_2_ Peak Test

A sub-sample of participants completed a maximal oxygen uptake test on a treadmill to measure $\dot{\text{V}}$O_2_ peak (ml kg^-1^ min^-1^). The speed of the test was constant and individualized for each participant based on the speed that corresponded with 85% HR_max_ during the prior submaximal test. The gradient of the treadmill increased by 1% per minute of the test completed. Participants were required to run to the point of volitional exhaustion, which was indicated by the participant’s rating of perceived exertion on a 6 – 20 Borg scale (Borg, 1998) in conjunction with live monitoring of their heart rate. Prior to the exercise laboratory tests, all participants were shown the Borg scale and given an age appropriate explanation of the information provided from this psychological evaluation of perceived exertion. Participants were instructed to point to the scale to indicate the rating relating to how intense the exercise felt when they were shown the scale. During the final minute of the test, participants breathed expired air into a Douglas Bag, which was later analyzed on a Servomex 1440 Gas Analyser (Servomex, United States) to calculate $\dot{\text{V}}$O_2_ peak (ml kg^-1^ min ^-1^). Verbal encouragement was provided throughout the test to ensure the participant worked to the point of volitional exhaustion.

### Statistical Analysis

An *a priori* power calculation was performed using GPower 3.1.9.2 and based on IL-6 data in previous research (Ischander et al., 2007), with an alpha probability level of 0.05, 4 groups and 1 covariate; a total sample size of 107 was required.

Participants were separated into distinct fitness quartiles quantified by distance ran on the MSFT, blood lactate concentration at 8.5 km h^-1^ and maximal oxygen uptake determined from the $\dot{\text{V}}$O_2_ peak test (Table 1). Adiposity quartiles were quantified from the sum of skinfolds. The first quartile (defined as the 25th percentile, which included participants with values ≤25% of all values in the present study) included participants with the lowest physical fitness and highest adiposity. The effect of physical fitness and adiposity quartile on risk factors for cardio-metabolic disease was analyzed via two-way between subjects ANCOVA with maturation (APHV) used as a covariate in SPSS (Version 24, SPSS Inc., Chicago, Il, United States). When significant interactions were observed between physical fitness/adiposity and sex, *post hoc* comparisons were performed using a least significant difference (LSD) correction. *Post hoc* comparisons interrogated significant interactions between boys and girls within quartiles and within sex comparisons across quartiles. Where significant effects existed, effect sizes were calculated as Cohen’s d. Multiple linear regression was used to examine the relationship (adjusted for APHV) between independent variables (distance on the MSFT, $\dot{\text{V}}$O_2_ peak and adiposity) and each cardio-metabolic risk factor (IL-6, IL-1β, IL-10, TNF-α, CRP, fasted blood glucose and plasma insulin, HOMA, systolic, diastolic and mean arterial blood pressure). Blood lactate concentration during sub-maximal exercise was not examined in the multiple linear regression, as the sample size did not meet the minimum criteria necessary for four predictor variables (Vanvoorhis and Morgan, 2007). For all analysis significance was accepted as *P* < 0.05 and data are presented as mean ± SEM.

**Table 1:** Performance in the multi-stage fitness test (distance run), $\dot{\text{V}}$O_2_ peak, Blood lactate at 8.5 km h^-1^ on the speed lactate test and adiposity from sum of skinfolds separated by sex and into quartiles.

Quartile	Distance run on the MSFT (m)		$\dot{\text{V}}$O_2_ Peak (ml kg^-1^ min^-1^)		Blood lactate at 8.5 km h^-1^ on the speed lactate test (mmol L^-1^)		Adiposity from sum of skinfolds (mm)	
	Boys	Girls	Boys	Girls	Boys	Girls	Boys	Girls
1	860 ± 60	480 ± 60*	40.2 ± 3.2	34.1 ± 1.5*	2.71 ± 0.17	5.20 ± 0.96*	56 ± 3	97 ± 3*
2	1300 ± 20	900 ± 40*	49.9 ± 0.6	42.9 ± 0.8*	2.30 ± 0.27	3.62 ± 0.79*	40 ± 1	54 ± 2*
3	1500 ± 20	1160 ± 20*	52.7 ± 0.4	48.9 ± 0.7*	1.95 ± 0.38	2.62 ± 0.54*	34 ± 1	39 ± 1*
4	1800 ± 40	1540 ± 60*	57.9 ± 1.2	58.0 ± 1.3	1.07 ± 0.22	1.61 ± 0.88	27 ± 1	29 ± 1

*Participants in quartile one had the lowest physical fitness or highest adiposity (Mean ± SEM). ^∗^Denotes significant differences between boys and girls in respective quartiles.*

## Results

Quartiles for each variable (distance ran on the MSFT, $\dot{\text{V}}$O_2_ peak, blood lactate concentration at 8.5 km h^-1^ and adiposity) were separately determined for boys and girls (Table 1). When considering the effect of sex on MSFT performance, boys ran further than their female counterparts across all quartiles (all *p* < 0.001). Similarly, boys in quartiles one to three had a higher peak oxygen consumption, a lower blood lactate concentration at 8.5 km h^-1^ and lower adiposity when compared with their female counterparts (all *p* < 0.001). There was no difference between boys and girls in $\dot{\text{V}}$O_2_ peak (*p* = 0.970) or adiposity (*p* = 0.086; Table 1) in quartile four. BMI had no statistically significant effect on any of the outcome variables (all *p* > 0.05).

### Inflammation

#### IL-6

**FIGURE 1:**
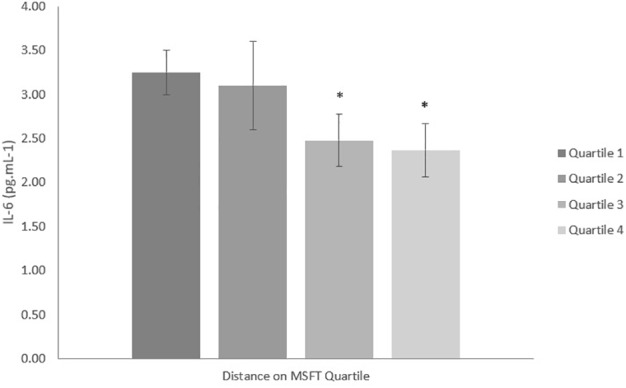
IL-6 concentration (pg mL^-1^) separated into quartiles by distance run on the multi-stage fitness test. Participants in quartile one covered the shortest distance. Mean ± SEM, main effect of training status *p* = 0.038. ^∗^denotes significant difference from quartile one.

When separating participants into quartiles based on distance ran on the MSFT, IL-6 concentration was higher in quartile one when compared with participants in the third (*p* = 0.011, *d* = 0.6) and fourth quartiles [*p* = 0.009, *d* = 0.7; main effect: *F*_(3,90)_ = 2.9, *p* = 0.038; Figure 1 and Table 2]. There was no difference in IL-6 concentration when separating participants by $\dot{\text{V}}$O_2_ peak, blood lactate concentration at 8.5 km h^-1^ or adiposity (all *p* > 0.05), nor was there any difference between boys and girls (main effect of sex: all *p* > 0.05; interaction effect: all *p* > 0.05). The multiple regression analysis (Table 3) revealed that distance ran on the MSFT was the only statistically significant predictor of IL-6 concentration, after adjustment for APHV, with a negative relationship observed between the two variables (β = -0.291, *p* = 0.031).

**Table 2:** Inflammatory cytokines (IL-6, IL-1β, IL-10, TNF-α) and CRP separated into quartiles determined from distance run on the multi-stage fitness test, blood lactate concentration at 8.5 km h^-1^ during the speed lactate test, $\dot{\text{V}}$O_2_ peak and adiposity (Mean ± SEM).

	Distance Run on the MSFT (m)				$\dot{\text{V}}$O_2_ Peak (ml kg^-1^ min^-1^)				Blood lactate at 8.5 km h^-1^ during speed lactate test (mmol L^-1^)				Adiposity from sum of skinfolds (mm)			
	Q1	Q2	Q3	Q4	Q1	Q2	Q3	Q4	Q1	Q2	Q3	Q4	Q1	Q2	Q3	Q4
IL-6 (pg mL^-1^)	3.25 ± 0.25	3.10 ± 0.50	2.48 ± 0.30^∗^	2.37 ± 0.30^∗^	3.76 ± 0.54	3.47 ± 0.29	2.60 ± 0.37	2.60 ± 0.32	3.57 ± 0.52	3.72 ± 0.57	2.70 ± 0.50	2.88 ± 0.53	3.05 ± 0.26	3.23 ± 0.35	2.95 ± 0.29	2.47 ± 0.29
IL-1β (pg mL^-1^)	4.78 ± 0.85	4.34 ± 0.47	2.96 ± 0.29^∗^	2.47 ± 0.29^∗^	4.67 ± 1.70	3.14 ± 0.63	3.16 ± 0.62	3.25 ± 0.71	7.35 ± 2.60	3.15 ± 0.65	2.72 ± 1.25	2.82 ± 0.68	5.51 ± 1.06	3.36 ± 0.39	3.58 ± 0.44	2.86 ± 0.34
IL-10 (pg mL^-1^)	1.80 ± 0.31	2.08 ± 0.19	2.41 ± 0.41	3.80 ± 0.77	2.27 ± 0.43	2.17 ± 0.23	2.18 ± 0.37	3.82 ± 1.23	1.65 ± 0.45	2.61 ± 0.48	2.96 ± 0.61	3.62 ± 0.45^∗^	2.18 ± 0.31	2.11 ± 0.31	2.97 ± 0.76	2.40 ± 0.38
TNF-α (pg mL^-1^)	1.93 ± 0.53	1.47 ± 0.20	1.47 ± 0.19	1.42 ± 0.24	1.71 ± 0.34	1.24 ± 0.15	1.59 ± 0.23	1.74 ± 0.32	2.32 ± 0.62	2.00 ± 0.57	1.21 ± 1.91	1.86 ± 0.42	1.89 ± 0.17	1.60 ± 0.26	1.65 ± 0.24	1.89 ± 0.54
CRP (mg L^-1^)	0.52 ± 0.14	0.47 ± 0.21	0.52 ± 0.31	0.35 ± 0.19	0.43 ± 0.14	0.45 ± 0.15	0.41 ± 0.16	0.30 ± 0.10	0.69 ± 0.21	0.68 ± 0.28	0.86 ± 0.39	0.45 ± 0.20	0.52 ± 0.14	0.47 ± 0.14	0.45 ± 0.16	0.38 ± 0.10

*^∗^denotes significantly different from quartile one.*

**Table 3:** Standardized regression summary for distance run on the MSFT, $\dot{\text{V}}$O_2_ peak, and adiposity with individual risk factors.

	Distance run on MSFT (m)			$\dot{\text{V}}$O_2_ peak test (ml kg^-1^ min^-1^)			Adiposity from sum of skinfolds (mm)		
	*R*^2^ adj.	β	*p*	*R*^2^ adj.	β	*p*	*R*^2^ adj.	β	*p*
IL-6 (pg mL^-1^)	0.085	–0.291	0.031*	0.035	0.060	0.800	–0.004	–0.005	0.978
IL-1β (pg mL^-1^)	0.164	–0.405	0.005*	0.244	0.313	0.106	0.004	0.004	0.981
IL-10 (pg mL^-1^)	0.108	0.325	0.021*	0.134	0.151	0.419	0.118	0.173	0.419
TNF-α (pg mL^-1^)	0.098	0.167	0.397	0.054	0.107	0.489	0.120	0.178	0.420
Blood glucose (mmol L^-1^)	0.297	–0.545	<0.001*	–0.113	–0.145	0.390	0.152	0.190	0.246
Plasma insulin (mU L^-1^)	–0.079	–0.097	0.563	–0.150	–0.172	0.269	0.266	0.515	<0.001*
HOMA	–0.096	–0.127	0.488	–0.105	–0.122	0.450	0.256	0.506	<0.001*
Systolic blood pressure (mmHg)	0.060	–0.091	0.666	–0.025	–0.102	0.855	0.31	0.142	0.825
Diastolic blood pressure (mmHg)	0.094	0.135	0.472	0.000	0.000	0.998	0.067	0.259	0.042*
Mean arterial pressure (mmHg)	0.115	0.163	0.383	–0.018	–0.023	0.892	0.088	0.332	0.011*

*^∗^denotes significant relationship.*

#### IL-1β

**FIGURE 2:**
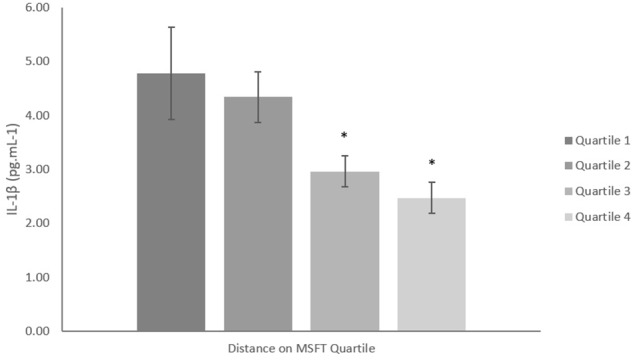
IL-1β concentration (pg mL^-1^) separated into quartiles by distance run on the multi-stage fitness test. Participants in quartile one covered the shortest distance. Mean ± SEM; main effect of training status *p* = 0.032. ^∗^denotes significant difference from quartile one.

When separating participants into quartiles based on distance ran on the MSFT, IL-1β concentration was higher in quartile one when compared with participants in the third (*p* = 0.039, *d* = 0.6) and fourth quartiles [*p* = 0.008, *d* = 0.8; main effect: *F*_(3,96)_ = 3.1, *p* = 0.032; Figure 2 and Table 2]. There was no difference in IL-1β concentration when separating participants by $\dot{\text{V}}$O_2_ peak, blood lactate concentration at 8.5 km h^-1^ or adiposity (all *p* > 0.05). When considering the effect of sex, IL-1β concentration was higher in boys than girls [boys; 4.26 ± 0.44 pg⋅mL^-1^, girls; 2.94 ± 0.45 pg⋅mL^-1^; main effect of sex: *F*_(1,96)_ = 4.4, *p* = 0.039, *d* = 0.4]. The effect of fitness or adiposity was not different between boys and girls (interaction: all *p* > 0.05). The multiple regression analysis (Table 3) revealed that distance ran on the MSFT was the only statistically significant predictor of IL-1β concentration, after adjustment for APHV, with a negative relationship observed between the two variables (β = -0.405, *p* = 0.005).

#### IL-10

**FIGURE 3:**
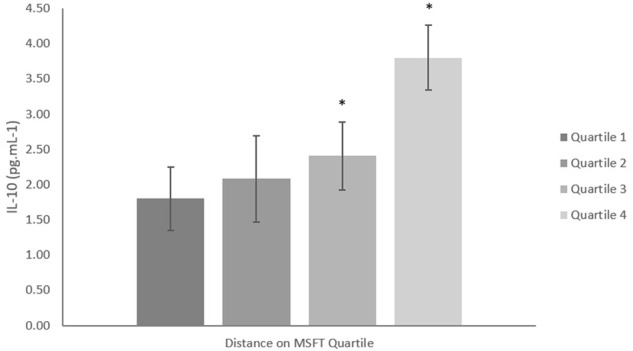
IL-10 concentration (pg mL^-1^) separated into quartiles by blood lactate concentration at 8.5 km h^-1^. Participants in quartile one had the lowest training status. Mean ± SEM, main effect of training status *p* = 0.035. ^∗^denotes significant difference from quartile one.

When separating participants into quartiles determined by blood lactate concentration at 8.5 km h^-1^, IL-10 concentration was lower in quartile one when compared with participants in quartile four [*p* = 0.006, *d* = 0.9; main effect: *F*_(3,27)_ = 3.6, *p* = 0.035, Figure 3 and Table 2]. There was no difference in IL-10 concentration when separating participants by distance ran on the MSFT, $\dot{\text{V}}$O_2_ peak or adiposity (all *p* > 0.05), nor was there any difference between boys and girls (main effect of sex: all *p* > 0.05; interaction: all *p* > 0.05). The multiple regression analysis (Table 3) revealed that distance ran on the MSFT was the only statistically significant predictor of IL-10 concentration, after adjustment for APHV, with a positive relationship observed between the two variables (β = 0.325, *p* = 0.021).

#### TNF-α and CRP

When separating participants into quartiles by distance covered on the MSFT, $\dot{\text{V}}$O_2_ peak, blood lactate concentration at 8.5 km h^-1^ and adiposity there was no difference in TNF-α or CRP concentration across quartiles (all *p* > 0.05, Table 2). Furthermore, there was no difference between boys and girls (main effect of sex: all *p* > 0.05; interaction: all *p* > 0.05). Multiple regression revealed no statistically significant predictors of TNF-α or CRP concentration (Table 3).

### Blood Glucose, Plasma Insulin Concentration and HOMA

#### Fasting Blood Glucose

When separating participants into quartiles determined by distance ran on the MSFT, blood glucose concentration was higher in quartile one when compared with quartile two (*p* = 0.025, *d* = 0.5), three (*p* < 0.001, *d* = 1.1) and four [*p* < 0.001, *d* = 1; main effect: *F*_(3,110)_ = 7.1, *p* < 0.001; Table 4]. When separating participants into $\dot{\text{V}}$O_2_ peak quartiles, blood glucose concentration was higher in quartile one when compared with participants in the fourth quartile [*p* = 0.001, *d* = 1.1; main effect: *F*_(3,68)_ = 3.9, *p* = 0.013; Table 4]. When considering the effect of sex there was no difference between boys and girls (main effect of sex: all *p* > 0.05; interaction: all *p* > 0.05). The multiple regression analysis (Table 3) revealed that distance ran on the MSFT was the only statistically significant predictor of blood glucose concentration, after adjustment for APHV, with a negative relationship observed (β = -0.545, *p* < 0.001).

**Table 4:** Cardio-metabolic risk factors including blood glucose, plasma insulin, HOMA and blood pressure separated into quartiles determined from distance run on the multi-stage fitness test, blood lactate concentration at 8.5 km h^-1^ during the speed lactate test, $\dot{\text{V}}$O_2_ peak, and adiposity (Mean ± SEM).

	Distance run on the MSFT (m)				$\dot{\text{V}}$O_2_ Peak (ml kg^-1^ min^-1^)				Blood lactate at 8^.^5 km h^-1^ during speed lactate test (mmol L^-1^)				Adiposity from Sum of Skinfolds (mm)			
	Q1	Q2	Q3	Q4	Q1	Q2	Q3	Q4	Q1	Q2	Q3	Q4	Q1	Q2	Q3	Q4
Blood Glucose (mmol L^-1^)	4.60 ± 0.44	4.35 ± 0.48^∗^	4.08 ± 0.44^∗^	4.11 ± 0.53^∗^	4.63 ± 0.58	4.32 ± 0.57	4.25 ± 0.49	4.00 ± 0.54^∗^	4.48 ± 0.52	4.32 ± 0.65	4.36 ± 0.78	3.82 ± 0.53	4.54 ± 0.68	4.27 ± 0.44	4.19 ± 0.56	4.20 ± 0.44^∗†^
Plasma Insulin (mU L^-1^)	8.99 ± 1.04	6.71 ± 0.76	5.60 ± 0.54^∗^	4.86 ± 0.82^∗^	8.49 ± 1.24	7.07 ± 0.66	5.18 ± 0.63^∗^	4.02 ± 0.54^∗†^	7.00 ± 0.87	5.60 ± 0.90	6.23 ± 0.67	3.51 ± 0.88^∗^	9.08 ± 1.07	5.55 ± 0.69^∗^	6.68 ± 0.74^∗^	5.70 ± 0.80^∗^
HOMA	2.00 ± 0.68	1.22 ± 0.18^∗^	1.21 ± 0.18^∗^	0.78 ± 0.18^∗^	1.81 ± 0.32	1.48 ± 0.18	0.90 ± 0.09^∗^	0.78 ± 0.13^∗†^	1.35 ± 0.14	1.61 ± 0.17	1.01 ± 0.13	0.60 ± 0.21	1.90 ± 0.27	1.14 ± 0.14^∗^	1.20 ± 0.15^∗^	1.01 ± 0.12^∗^
Systolic blood pressure (mmHg)	112 ± 2	112 ± 2	111 ± 2	111 ± 2	111 ± 2	108 ± 2	110 ± 2	110 ± 3	104 ± 2	111 ± 2	106 ± 2	113 ± 3	115 ± 2	109 ± 2	112 ± 2	110 ± 2
Diastolic blood pressure (mmHg)	69 ± 2	67 ± 1	70 ± 1	73 ± 1	70 ± 2	72 ± 2	68 ± 1	65 ± 1	74 ± 1	70 ± 2	66 ± 1	68 ± 1	73 ± 1	72 ± 2	67 ± 1^∗^	69 ± 1^∗^
Mean arterial pressure (mmHg)	86 ± 2	84 ± 1	82 ± 1	83 ± 1	83 ± 2	84 ± 1	82 ± 1	80 ± 1	84 ± 1	83 ± 2	79 ± 2	83 ± 1	87 ± 1	83 ± 1^∗^	82 ± 1^∗^	83 ± 1^∗^

*^∗^denotes significantly different from quartile one; ^†^significantly different from quartile two.*

When separating participants into adiposity quartiles, blood glucose concentration was higher in quartile one when compared with participants in quartile four [p = 0.012, *d* = 0.6; main effect: *F*_(3,115)_ = 3.0, *p* = 0.035; Table 4]. Participants in quartile two also had higher blood glucose concentration when compared with quartile four (second quartile; *p* = 0.011, *d* = 0.5). There was no difference in blood glucose concentration across all quartiles between boys and girls (main effect of sex: *p* = 0.637). There was an effect of adiposity on sex [interaction: *F*_(3,115)_ = 3.4, *p* = 0.019], in that girls in the first quartile had higher blood glucose concentration (4.81 ± 0.59 mmol L^-1^) when compared with quartiles two (4.12 ± 0.44 mmol L^-1^, *p* = 0.001, *d* = 1.3), third (4.23 ± 0.52 mmol L^-1^, *p* = 0.004, *d* = 1) and four (4.14 ± 0.46 mmol L^-1^, *p* = 0.001, *d* = 1.3). There was no difference in blood glucose concentration across adiposity quartiles in boys (all *p* > 0.05).

#### Fasting Plasma Insulin

When separating participants into quartiles determined by distance ran on the MSFT, plasma insulin concentration was higher in quartile one when compared with participants in quartiles three (*p* = 0.005, *d* = 0.8) and four [*p* < 0.001, *d* = 1; main effect: *F*_(3,102)_= 5.5, *p* = 0.002; Table 4]. When separating participants into quartiles determined by $\dot{\text{V}}$O_2_ peak, plasma insulin concentration was higher in participants in quartile one when compared with participants in quartiles three (*p* = 0.009, *d* = 0.7) and four [*p* < 0.001, *d* = 1; main effect: *F*_(3,62)_ = 5.8, *p* = 0.002; Table 4]. Participants in quartile two also had higher plasma insulin concentrations when compared with quartile four (*p* = 0.009, *d* = 0.7). When separating participants into quartiles determined from blood lactate concentration at 8.5 km h^-1^, plasma insulin concentration was higher in quartile one when compared with participants in quartile four [*p* = 0.012, *d* = 0.9; main effect: *F*_(3,28)_ = 3.8, *p* = 0.043; Table 3]. When considering the effect of sex, plasma insulin concentration was higher in girls (7.73 ± 0.58 mU L^-1^) than boys [boys; 6.05 ± 0.55 mU L^-1^; main effect of sex: *F*_(1,101)_ = 4.4, *p* = 0.037, *d* = 0.4]; yet, the effect of fitness did not differ between boys and girls (interaction: all *p* > 0.05).

When separating participants into quartiles determined by adiposity, plasma insulin concentration was higher in quartile one when compared with participants in quartiles two (*p* = 0.003, *d* = 0.9), three (*p* = 0.044, *d* = 0.6) and four [*p* = 0.004, *d* = 0.8; main effect: *F*_(3,105)_ = 4.0, *p* = 0.010; Table 4]. When considering the effect of sex, plasma insulin concentration was higher in girls [7.59 ± 0.56 mU L^-1^ vs. 5.86 ± 0.57 mU L^-1^; main effect of sex: *F*_(1,105)_ = 4.7, *p* = 0.033, *d* = 0.4]. There was an effect of adiposity on sex [interaction: *F*_(3,105)_ = 3.5, *p* = 0.018], in that girls having the highest adiposity had higher plasma insulin concentrations than boys in the same quartile [boys; 6.15 ± 0.82 mU L^-1^, girls; 11.81 ± 1.67 mU L^-1^, *F*_(1,97)_ = 12.9, *p* < 0.001, *d* = 1]. Girls in quartile one had increased plasma insulin concentration when compared with girls in quartiles two (6.56 ± 0.86 mU L^-1^, *p* < 0.001, *d* = 1.2) three (6.85 ± 0.57 mU L^-1^, *p* = 0.002, *d* = 1.1) and four (5.11 ± 0.57 mU L^-1^, *p* < 0.001, *d* = 1.4). There was no difference in plasma insulin concentration in boys across quartiles (all *p* > 0.05). The multiple regression analysis (Table 3) revealed that adiposity was the only statistically significant predictor of plasma insulin concentration, after adjustment for APHV, with a positive relationship observed between the two variables (β = 0.515, *p* < 0.001).

#### HOMA

When separating participants into quartiles determined by distance ran on the MSFT, HOMA was higher in quartile one when compared with participants in quartiles two (*p* = 0.002, *d* = 0.8), three (*p* = 0.002, *d* = 0.8) and four [*p* < 0.001, *d* = 1.4; main effect: *F*_(3,101)_ = 9.4, *p* < 0.001; Table 4]. When separating participants into fitness quartiles determined by $\dot{\text{V}}$O_2_ peak, HOMA was higher in quartile one when compared with participants in quartiles three (*p* = 0.003, *d* = 0.8) and four [*p* = 0.001, *d* = 1.1; main effect: *F*_(3,60)_ = 5.7, *p* = 0.002; Table 3]. Participants in quartile two also had increased HOMA when compared with participants in quartile four (*p* = 0.019, *d* = 0.7). When considering the effect of sex, HOMA was higher in girls (1.50 ± 0.13) than boys [1.14 ± 0.12; main effect of sex: *F*_(1,99)_ = 4.1, *p* = 0.046, *d* = 0.4], yet the effect of VO_2_ peak on HOMA did not differ between boys and girls (all *p* > 0.05). When separating participants into quartiles by blood lactate concentration at 8.5 km h^-1^ there was no difference in HOMA across quartiles (all *p* > 0.05, Table 4).

When separating participants into quartiles determined by adiposity, HOMA was higher in quartile one when compared with participants in quartiles two (*p* = 0.002, *d* = 0.9), three (*p* = 0.005, *d* = 0.8) and four [*p* < 0.001, *d* = 1; main effect: *F*_(3,103)_ = 5.6, *p* = 0.001; Table 4]. When considering the effect of sex, HOMA was higher in girls [1.52 ± 0.12 vs. 1.10 ± 0.12; main effect of sex: *F*_(1,103)_ = 5. 9, *p* = 0.017, *d* = 0.5]. There was also an effect of adiposity on sex [interaction: *F*_(3,103)_ = 4.0, *p* = 0.010, *d* = 1.5], in that girls in quartile one had higher HOMA (2.58 ± 0.44) than their male counterparts (1.22 ± 0.19). Girls with the highest adiposity also had increased HOMA when compared with girls in quartiles two (1.36 ± 0.19, *p* = 0.001, *d* = 0.9), third (1.18 ± 0.25, *p* < 0.001, *d* = 1.1) and four (0.94 ± 0.15, *p* < 0.001, *d* = 1.3). There was no difference in HOMA across adiposity quartiles in boys (all *p* > 0.05). The multiple regression analysis (Table 3) revealed that adiposity was the only statistically significant predictor of HOMA, after adjustment for APHV, with a positive relationship observed between the two variables (β = 0.506, *p* < 0.001).

### Blood Pressure

#### Systolic Blood Pressure

When separating participants into quartiles based on distance ran during the MSFT, $\dot{\text{V}}$O_2_ peak and blood lactate concentration at 8.5 km h^-1^ during the speed lactate test or adiposity, there was no difference in systolic blood pressure (all *p* > 0.05, Table 4). When considering the effect of sex there was no difference in systolic blood pressure between boys and girls (main effect of sex: all *p* > 0.05; interaction: all *p* > 0.05). The regression model for systolic blood pressure identified no statistically significant predictors.

#### Diastolic Blood Pressure

When separating participants into adiposity quartiles, diastolic blood pressure was higher in quartile one when compared with participants in quartiles three (*p* = 0.003, *d* = 0.7) and four [*p* = 0.046, *d* = 0.5; main effect: *F*_(3,116)_ = 3.3, *p* = 0.023; Table 4]. There was no difference in diastolic blood pressure across quartiles when participants were separated by distance ran during the MSFT, $\dot{\text{V}}$O_2_ peak and blood lactate concentration at 8.5 km h^-1^ during the speed lactate test (all *p* > 0.05), nor was there any difference between boys and girls (main effect of sex: all *p* > 0.05; interaction: all *p* > 0.05). The multiple regression analysis (Table 3) revealed that adiposity was the only statistically significant predictor of diastolic blood pressure, after adjustment for APHV, with a positive relationship between the two variables (β = 0.259, *p* = 0.042).

#### Mean Arterial Pressure

When separating participants into adiposity quartiles, mean arterial pressure was higher in quartile one when compared with quartiles two (*p* = 0.021, *d* = 0.6), three (*p* = 0.004, *d* = 0.7) and four [*p* = 0.017, *d* = 0.6; main effect: *F*_(3,116)_ = 3.5, *p* = 0.018; Table 4]. There was no difference in mean arterial pressure when participants were separated by distance ran during the MSFT, $\dot{\text{V}}$O_2_ peak or blood lactate concentration at 8.5 km h^-1^ during the speed lactate test (all *p* > 0.05), nor was there any difference between boys and girls (main effect of sex: all *p* > 0.05; interaction: all *p* > 0.05). The multiple regression analysis (Table 3) revealed that adiposity was the only statistically significant predictor of mean arterial pressure, after adjustment for APHV, with a positive relationship observed between the two variables (β = 0.322, *p* = 0.011).

## Discussion

The primary finding of the present study was that adolescents categorized below the 25th centile for distance ran on the MSFT in the current dataset exhibited increased concentrations of pro-inflammatory cytokines IL-6 and IL-1β and reduced concentrations of the anti-inflammatory mediator IL-10 when compared with those categorized above the 25th centile. The present study is the first to report that distance ran on the MSFT and the blood lactate response to exercise were the only measures to influence inflammatory cytokine concentrations in adolescents, both of which are deemed more sensitive measures of an individual’s physical capacity to perform prolonged exercise. In addition, the multiple regression revealed that the MSFT was the only significant predictor of inflammation in adolescents (with no relationship observed for $\dot{\text{V}}$O_2_ peak or adiposity). Furthermore, adolescents categorized below the 25th percentile with the lowest distance ran on the MSFT and $\dot{\text{V}}$O_2_ peak exhibited increased metabolic risk factors (including fasted blood glucose, plasma insulin and HOMA), whilst adolescents with the highest adiposity also presented with increased diastolic and mean arterial blood pressure compared to adolescents in all other quartiles. These findings emphasize the importance of enhancing the physical capacity to perform prolonged exercise, as evidenced by performance on the MSFT, and maintaining a healthy body composition during adolescence in order to attenuate the risk of developing early onset cardiovascular disease and type 2 diabetes.

Adolescents with the lowest MSFT performance in the present study exhibited increased concentrations of pro-inflammatory cytokines IL-6 and IL-1β, and reduced concentrations of anti-inflammatory mediator IL-10 in comparison to adolescents in all other quartiles. These findings are novel as the present study is the first to measure a range of inflammatory cytokines that are reflective of low-grade chronic inflammation in a heterogeneous population of male and female adolescents (Gleeson et al., 2011). The finding that adolescents with the lowest physical fitness have increased concentrations of pro-inflammatory mediators is consistent with previous studies, in that increased concentrations of IL-6 (Bugge et al., 2012; Buchan et al., 2015) and CRP (Buchan et al., 2015) are observed in participants in the lowest quartile for physical fitness. However, the present study is the first to report that participants with the lowest MSFT performance have reduced circulating levels of IL-10. These findings are in contrast to those of Jürimäe et al. (2017b) whereby IL-10 concentration was similar in female rhythmic gymnasts and untrained controls. These discrepant findings might relate to the different methods used to categorize participants, as the present study measured the participant’s physical capacity to perform prolonged exercise and body composition, whereas Jürimäe et al. (2017b) categorized participants solely based on participation in rhythmic gymnastics or not. Therefore, the present study assessed the objective relationship between performance in submaximal and maximal exercise tests and anti-inflammatory mediator IL-10. Increased concentrations of IL-10 protect against risk factors for cardio-metabolic diseases, as *in vitro* studies report that IL-10 inhibits the synthesis of IL-1β and TNF-α which promote the development of low-grade chronic inflammation (Petersen and Pedersen, 2005). As acute bouts of physical activity transiently increase IL-10 concentrations (Petersen and Pedersen, 2005), it is not surprising that participants with the lowest performance on the MSFT had significantly reduced concentrations of the potent anti-inflammatory mediator. Furthermore, there were no differences between pro- or anti-inflammatory cytokine concentrations in adolescents categorized above the 25th percentile, which is consistent with previous research (Buchan et al., 2015). These findings suggest that an enhanced physical capacity to perform prolonged exercise protects against low-grade chronic inflammation in adolescents by reducing exposure to pro-inflammatory mediators and increasing systemic concentrations of the anti-inflammatory cytokine IL-10.

Performance on the MSFT and the blood lactate response to submaximal exercise were the only measures to influence inflammation in adolescents in the present study. This finding was also observed in the multiple regression model, which revealed that distance ran on the MSFT was the only significant predictor of inflammation in adolescents, whilst $\dot{\text{V}}$O_2_ peak and adiposity were not related to inflammation. Previous studies that assessed physical fitness based on the $\dot{\text{V}}$O_2_ peak test (Ischander et al., 2007; Steene-Johannessen et al., 2013) also reported no relationship between pro-inflammatory mediators (IL-6, TNF-α, and CRP) and physical fitness in adolescents. In contrast, inverse associations between pro-inflammatory mediators (IL-6, TNF-α, and CRP) and physical fitness have been observed when performance on the MSFT was the preferred measure of physical fitness (Silva et al., 2014; Buchan et al., 2015).

To the authors’ knowledge, the present study is the first to have considered that the methodology used to measure an individual’s capacity to perform prolonged exercise influences the relationship between physical fitness and risk factors for cardio-metabolic diseases in adolescents. The acute anti-inflammatory response stimulated post-exercise reduces low-grade chronic inflammation in adolescents if repeated regularly (Mendham et al., 2015). Increased engagement with regular physical activity improves exercise tolerance and initiates peripheral adaptations in the muscle, including enhanced efficiency of mitochondrial biogenesis, increased fat oxidation and reduced blood lactate concentration at a given exercise intensity (Joyne and Carsten, 2018). The MSFT and blood lactate response to sub-maximal exercise measure such peripheral changes and are therefore considered to be sensitive to changes in the ability to perform prolonged exercise. In contrast, the $\dot{\text{V}}$O_2_ peak test is limited when measuring peripheral adaptations as it is predominantly determined by central systems (cardiovascular and respiratory) that have a strong genetic predisposition (Joyne and Carsten, 2018). Consequently, the MSFT and blood lactate response to sub-maximal exercise are better suited in the measurement of physical fitness specifically for metabolic risk in young people. These findings suggest that adolescents can reduce low-grade chronic inflammation by enhancing performance on the MSFT, and that improving the capacity to perform prolonged exercise is a potential therapeutic intervention to prevent the development of risk factors for cardio-metabolic diseases.

In the present study, adolescents categorized below the 25th centile for distance ran on the MSFT, $\dot{\text{V}}$O_2_ peak and adiposity exhibited increased blood glucose and plasma insulin concentrations, and HOMA when compared with adolescents in all other quartiles. The participants categorized below the 25th centile for HOMA, the chosen measure of insulin resistance in the present study, were above the reference cut off values for insulin resistance (>1.65 in girls and >1.9 in boys) in healthy young people (Rocco et al., 2011). Whereas, participants categorized above the 25th centile were below the reference cut off values for insulin resistance. These findings are in conjunction with Silva et al. (2014) whereby increased adiposity and reduced maximal oxygen uptake increased metabolic risk (calculated from traditional risk factors including; blood pressure, blood glucose, triglyceride and HDL cholesterol) in adolescents. The multiple regression model in the current study showed that adiposity was the best predictor for metabolic risk factors (plasma insulin and HOMA) and blood pressure in adolescents. This finding is consistent with previous research in adults, which reported the sum of skinfold thickness to be the strongest predictor of insulin resistance (Abate et al., 1995). Studies in rodents reported that increasing adiposity drives an influx of free fatty acids, which deactivate insulin receptors and reduce insulin sensitivity (Capurso and Capurso, 2012). The findings of the present study indicate that adolescents, categorized with the highest adiposity are more insulin resistant than participants with lower adiposity in all other quartiles. Similarly, Paradis et al. (2004) observed an association between adiposity and both systolic and diastolic blood pressure in adolescents, yet the present study is the first to report the association between adiposity and mean arterial pressure in this population. The mechanisms relating adiposity with higher blood pressure in young people are relatively unknown, with disturbances in autonomic function being a potential mechanism (Paradis et al., 2004). These findings emphasize the importance of maintaining a healthy body composition in conjunction with enhancing physical performance to reduce the presence of risk factors for cardio-metabolic diseases in adolescents and slow the progression of chronic diseases, such as type 2 diabetes.

The present study also reports that girls with the highest adiposity had elevated plasma insulin concentration and reduced insulin sensitivity (HOMA) when compared with their male counterparts (boys categorized below the 25th centile for adiposity). These findings may be explained by the significantly increased adiposity of girls in quartile one when compared with boys and girls in all other quartiles (Table 1). These findings also support previous studies, which have reported that girls exhibited reduced postprandial insulin sensitivity when compared with boys of the same chronological age (Cooper et al., 2017). However, there was no difference in APHV between boys and girls in the present study and APHV was a covariate in the analysis to account for the potential confounding effects of maturation. Therefore, it is not feasible to suggest that the increased adiposity and insulin resistance observed in the girls in the present study was the result of differences in pubertal development. However, the potential confounding effect of puberty on the relationship between adiposity and insulin resistance in both males and females does warrant further research. Regardless of the mechanisms involved, it is apparent that adolescent girls with increased adiposity exhibit reduced insulin sensitivity when compared with their male counterparts. As such, future interventions should focus on promoting healthy body composition and physical fitness in adolescent girls, as the findings of the present study report that both variables can mediate improvements in insulin sensitivity.

The present study has several strengths including the measurement of a comprehensive panel of inflammatory cytokines in a heterogeneous sample of adolescents with diverse endurance capacities and adiposity, which allowed for the relationship between these variables and cardio-metabolic health to be determined. The heterogeneity of performance capacity (measured using the distance ran in the MSFT) in the present study ranged from the 30th–95th percentile for boys and the 20th–95th percentile for girls, when compared with normative data in European adolescents (Tomkinson et al., 2018). Similarly, the adiposity of the adolescents in the present study ranged from the 5th to >95th percentile for BMI, which also supports the heterogeneity of the participants recruited to the present study. The diversity of the adolescents analyzed in the present study allows for broad dissemination of the main findings of the study. However, a limitation to the present study was the lack of power for the blood lactate response to submaximal treadmill running and thus its exclusion from the multiple regression model. Therefore, future research should determine more fully the effect of the blood lactate response to exercise (as a measure of adolescent training status) on adolescent cardio-metabolic health. Further limitation include the absence of measurements pertaining to the ethnicity, daily dietary habits and the typical physical activity levels of the participants. Each of these measures are potential confounders in the relationship between performance tests and the risk factors measured in the present study (Hardman and Stensel, 2009). Nevertheless, given the difficulties of data collection in this age group and population the present study is the most comprehensive yet to examine fitness and the risk factors for cardio-metabolic disease in adolescents.

## Conclusion

In conclusion, the present study shows that a higher ability to perform prolonged exercise (as indicated by performance on the MSFT) in adolescents protects against the development of cardio-metabolic risk indicators that increase the likelihood of early onset cardiovascular disease and type 2 diabetes. These findings suggest that all young people can benefit from enhancing their ability to perform prolonged exercise as evidenced by the differences across quartiles based on MSFT performance. Furthermore, these findings are particularly important for those categorized below the 25th centile, as the benefits for metabolic risk factors were observed for those categorized above the 25th centile and for markers of inflammation for those above the 50th centile based on distance ran on the MSFT. Although there were also benefits of a high $\dot{\text{V}}$O_2_ peak and low adiposity, these were not as marked as the benefits of enhanced performance on the MSFT. Thus, enhancing performance on the MSFT is a key factor in successfully reducing cardio-metabolic risk in young people and thus training interventions should be given substantial attention in public policy interventions for young people.

## Ethics Statement

This study was carried out in accordance with the recommendations of Nottingham Trent University’s Ethical Advisory Committee with written informed consent and assent from all subjects. All subjects gave written informed consent in accordance with the Declaration of Helsinki. The protocol was approved by the Nottingham Trent University Ethical Advisory Committee.

## Author Contributions

KD, SC, CS, JM, and MN designed the study and collected the data. KD, GF, and AP analyzed the data. All authors contributed to interpretation of the data, drafting and revision of the manuscript, and approved the final version of the manuscript.

## Conflict of Interest Statement

The authors declare that the research was conducted in the absence of any commercial or financial relationships that could be construed as a potential conflict of interest.
